# Editorial for the Special Issue “Research on Radiotracers and Novel Radiopharmaceuticals for Cancer Therapy and Diagnosis”

**DOI:** 10.3390/pharmaceutics18060663

**Published:** 2026-05-27

**Authors:** Antonio Shegani

**Affiliations:** Institute of Nuclear and Radiological Sciences and Technology, Energy & Safety, NCSR “Demokritos”, 15310 Athens, Greece; ashegani@rrp.demokritos.gr

Cancer remains one of the most demanding health challenges worldwide, not only because of its high incidence and mortality, but also because of its biological heterogeneity, capacity for metastatic dissemination and frequent evolution under therapeutic pressure. In this context, molecular imaging and radiopharmaceutical therapy have become increasingly important components of precision oncology. Radiotracers can non-invasively interrogate disease biology in vivo, support patient stratification, guide therapeutic decisions, assess target expression across primary and metastatic lesions, and provide quantitative information that cannot be readily obtained through tissue sampling alone. In parallel, therapeutic radiopharmaceuticals and theranostic platforms have created new opportunities to combine target recognition, radionuclide selection and individualized treatment planning within a single conceptual framework.

The Special Issue “Research on Radiotracers and Novel Radiopharmaceuticals for Cancer Therapy and Diagnosis” was launched to highlight advances in the design, radiolabeling, biological evaluation and translational development of radiotracers and radiopharmaceuticals for oncology. Particular emphasis was placed on original research and review contributions addressing novel molecular targets, improved targeting vectors, preclinical models, radiochemical strategies, imaging methodology, pharmacokinetic optimization and approaches that may facilitate clinical translation. The accepted articles collectively reflect the breadth of current radiopharmaceutical research, spanning peptide-based PET probes, SPECT imaging agents for receptor phenotyping, and structural modification strategies to improve the metabolic stability of biologically active radioligands.

A major theme emerging from this Special Issue is the continued importance of receptor-targeted molecular imaging in breast cancer. Ebrahimi et al. reported the synthesis, gallium-68 labeling and preclinical evaluation of a DOTA-conjugated VEGFR1/2-targeting peptide for PET imaging of breast cancer (contribution 1). The radiotracer [^68^Ga]Ga-DOTA-Ahx-VGB3 was obtained with high radiochemical purity and demonstrated favorable in vitro stability. Its hydrophilic character was reflected in predominantly renal clearance, while cell-based studies in 4T1 breast cancer cells confirmed receptor-associated binding and internalization. In tumor-bearing mice, the compound accumulated rapidly at the tumor site, with tumor uptake reduced by blocking with the unlabeled peptide, supporting target-mediated accumulation. Although additional optimization of affinity, tumor retention and tumor-to-background contrast may be required, this study illustrates the potential of VEGFR-targeted peptide radiotracers for non-invasive visualization of angiogenesis-associated molecular features in breast cancer.

The clinical relevance of receptor imaging was further addressed by Bragina et al., who evaluated quantitative approaches for assessing HER2 expression in breast cancer using the scaffold protein [^99m^Tc]Tc-ADAPT6 (contribution 2). HER2 remains a central biomarker for treatment selection, yet spatial and temporal heterogeneity between primary tumors and metastatic sites can complicate clinical decision-making. In this prospective clinical study, SPECT/CT imaging was performed in treatment-naïve breast cancer patients with primary tumors and metastatic axillary lymph nodes. The authors compared imaging-derived parameters with HER2 status determined by immunohistochemistry and/or in situ hybridization. Among the evaluated metrics, SUV_max_ provided the best discrimination between HER2-positive and HER2-negative lesions, both in primary tumors and metastatic lymph nodes. The study also examined lesion-to-reference tissue ratios, including spleen-based ratios, which may retain practical value in settings where fully quantitative SPECT/CT is not available. This work addresses a key translational question: how radionuclide imaging can be used as a quantitative biomarker assay for target expression in patients.

A complementary direction was presented by Guarrochena et al., who investigated the amide-to-triazole switch in somatostatin-14-based radioligands as a strategy to improve metabolic stability while preserving receptor affinity (contribution 3). Structural modifications that improve metabolic stability may simultaneously alter excretion, metabolite profile and normal-organ retention. The established clinical success of somatostatin receptor-targeted radiopharmaceuticals has largely relied on metabolically stabilized analogs with preferential affinity for SST2R. However, broader somatostatin receptor targeting remains of interest for tumors with heterogeneous or non-SST2R-dominant receptor profiles. In this study, selected amide bonds in a DOTA-conjugated somatostatin-14-based radioligand were replaced by 1,4-disubstituted 1,2,3-triazole bioisosteres. Among the investigated analogs, [^111^In]In-XG1 preserved nanomolar affinity for several somatostatin receptor subtypes and showed improved in vivo stability compared with the parent compound. The work also demonstrated neprilysin’s involvement in radioligand metabolism, as pretreatment with Entresto^®^ increased the fraction of intact radioligand in blood. However, high renal uptake limited imaging performance, most likely because of the formation and renal retention of cationic radiometabolites.

Together, the contributions underscore several principles that are central to contemporary radiopharmaceutical development. First, molecular target selection remains decisive. VEGFR, HER2 and somatostatin receptors represent distinct biological systems, but each illustrates how target expression can be exploited for imaging, stratification or future therapeutic development. Second, the targeting vector must be optimized not only for affinity, but also for internalization, metabolic stability, route of clearance, dosimetry-relevant organ uptake and achievable tumor-to-background ratios. Third, quantitative imaging endpoints are becoming increasingly important, especially when radiopharmaceuticals are intended to support patient selection or biomarker assessment rather than solely for visual lesion detection. Finally, translational relevance depends on the integration of radiochemistry, pharmacology, imaging science, pathology and clinical workflow.

This field is now moving toward more refined radiopharmaceutical systems, including multi-receptor-targeting agents, optimized peptide and scaffold protein platforms, improved chelator–linker architectures, strategies to modulate renal and hepatic uptake, and theranostic pairs that bridge diagnostic imaging with radionuclide therapy. At the same time, clinical translation will require robust quality control, reproducible radiolabeling, standardized imaging protocols and clinically meaningful quantitative thresholds. Studies such as those included in this Special Issue contribute to this process by identifying both promising candidates and the limitations that must be addressed before broader clinical implementation.

I want to thank all authors who contributed to this Special Issue and the reviewers whose evaluations helped improve the quality and clarity of the published work. We also acknowledge the editorial and production teams of *Pharmaceutics* for their support throughout the preparation and completion of this Special Issue. We hope that this collection will stimulate further research into novel radiotracers, targeted radiopharmaceuticals and translational imaging approaches for cancer diagnosis and therapy.

